# A single-pieced, fully air-driven, cuff-inserted pseudo-blood pressure generator for on-site pre-screening test of non-invasive blood pressure monitor by nurses

**DOI:** 10.1186/s12938-019-0719-1

**Published:** 2019-10-16

**Authors:** Young Jun Hwang, Gun Ho Kim, Sung Uk Yun, Kyoung Won Nam

**Affiliations:** 1 Department of Biomedical Engineering, School of Medicine, Pusan National University, Yangsan, South Korea; 2 Interdisciplinary Program in Biomedical Engineering, School of Medicine, Pusan National University, Yangsan, South Korea; 3 Department of Biomedical Engineering, Pusan National University Yangsan Hospital, Yangsan, South Korea

**Keywords:** Blood pressure, Investigation, Portable, Patient safety

## Abstract

**Background:**

It is crucial to frequently inspect the proper operation of non-invasive electronic blood pressure monitors in various sites to prevent accidents from inaccurate blood pressure measurements, especially for large-scale hospitals. However, most conventional blood pressure monitor inspection devices are not suitable for such on-site investigation purpose. In this study, we propose a new single-pieced, fully air-driven pseudo blood pressure generator that is suitable for frequent on-site pre-screening tests of the blood pressure monitor by nurses.

**Results:**

The proposed model comprises a rigid cylindrical body, two simulated brachial arteries, two air-pumps, an electronic controller, and a pressure sensor. Control algorithm based on polynomial curve fitting was implemented to generate various user-instructed systolic blood pressure and heart-rate conditions automatically. To evaluate the performance and clinical feasibility of the proposed model, various experiments were performed using ten commercial electronic blood pressure monitors. Experimental results demonstrated that the values of the Pearson coefficient between the reference pseudo-blood pressure waveforms and the actually generated pressure waveforms were 0.983, 0.983 and 0.997 at 60, 70 and 80 beats/min, respectively (*p* < 0.05). Besides, during the experiments using ten commercial blood pressure monitors, the maximum error in average systolic blood pressure was 2.9 mmHg, the maximum standard deviation in average systolic blood pressure was 3.5 mmHg, and the maximum percentage error in average pumping rate was 3.2%, respectively.

**Conclusions:**

We expect that the proposed model can give an easy and comprehensive way for frequent on-site investigations of the blood pressure monitors by nurses, and improve the safety of patients with abnormal blood pressure, especially in most large-scale hospitals.

## Background

Blood pressure (BP) is a fundamental physiological signal to understand the physical status of patients with various symptoms and diseases; therefore, most hospitals repetitively gather BP information using non-invasive electronic BP monitors whenever the patient visits [1]. When the accuracy of in-hospital BP monitors deteriorates below the clinically permissible level during long-term use, several problems can occur. For example, a misdiagnosis as abnormal BP causes unnecessary time and money expense, and risk of improper medical treatment for normal BP individuals. In the case of misdiagnosis as normal BP, individuals who need urgent attention for abnormal BP can miss the time for proper medical treatment. Therefore, it is vital to maintain the appropriate operation of in-hospital BP monitors for the safety of patients with abnormal BP [2, 3].

In large-scale hospitals (with hundreds or thousands beds), experts from the engineering department should periodically examine all of the BP monitors in the hospital using inspection devices; when the estimated error is beyond the permitted limit, they send it to the authorized inspection agency for fine-tuning. For this case, the best way to guarantee the proper operation of BP monitors is to inspect each device more frequently (e.g., weekly or monthly). However, in most large-scale hospitals, there are too many devices to manage (i.e., inspect, tune, and repair) compared to the number of staffs in the engineering department. For example, seven engineering staffs should manage over 4000 devices in our hospital; among them, the number of BP monitors is over one hundred. As a result, the interval of periodic BP monitor inspection is generally once per a year, which may not sufficient to guarantee the proper operation because there are many error sources in actual circumstances such as scratch in the cuff, error in embedded pressure sensor or other electronic parts, and degradation of mechanical parts due to aging.

To improve the quality of BP monitor maintenance in large-scale hospitals, it is necessary to encourage the nurses to frequently perform on-site pre-screening tests by themselves at various sites such as wards, outpatient clinics, and emergency room, and ask the engineering staffs for further inspection only when the accuracy of tested device is suspicious. However, the price of conventional inspection devices is generally high to equip plural devices at all needed sites. In addition, inspection protocol of conventional devices is somewhat complicated for untrained nurses; e.g., open the external case of BP monitor, connect an air-tube inside the inspection device to the BP monitor, output pre-determined pressure vibrations via the air-tube, disconnect air-tube from the BP monitor, and close the external case. To encourage such self-screening tests by nurses, it is required to develop a new inspection tool suitable for such purpose that is (1) comprehensive and straightforward for untrained nurses, (2) small, light and single-pieced to improve portability and user convenience, and (3) relatively cheap to manufacture.

In this study, we propose a new technical model suitable for on-site pre-screening tests that can generate various pseudo-BP conditions through the cuff-inserted virtual brachial arteries, and verified the feasibility of the proposed model using ten BP monitors.

## Results

**Fig. 1:**
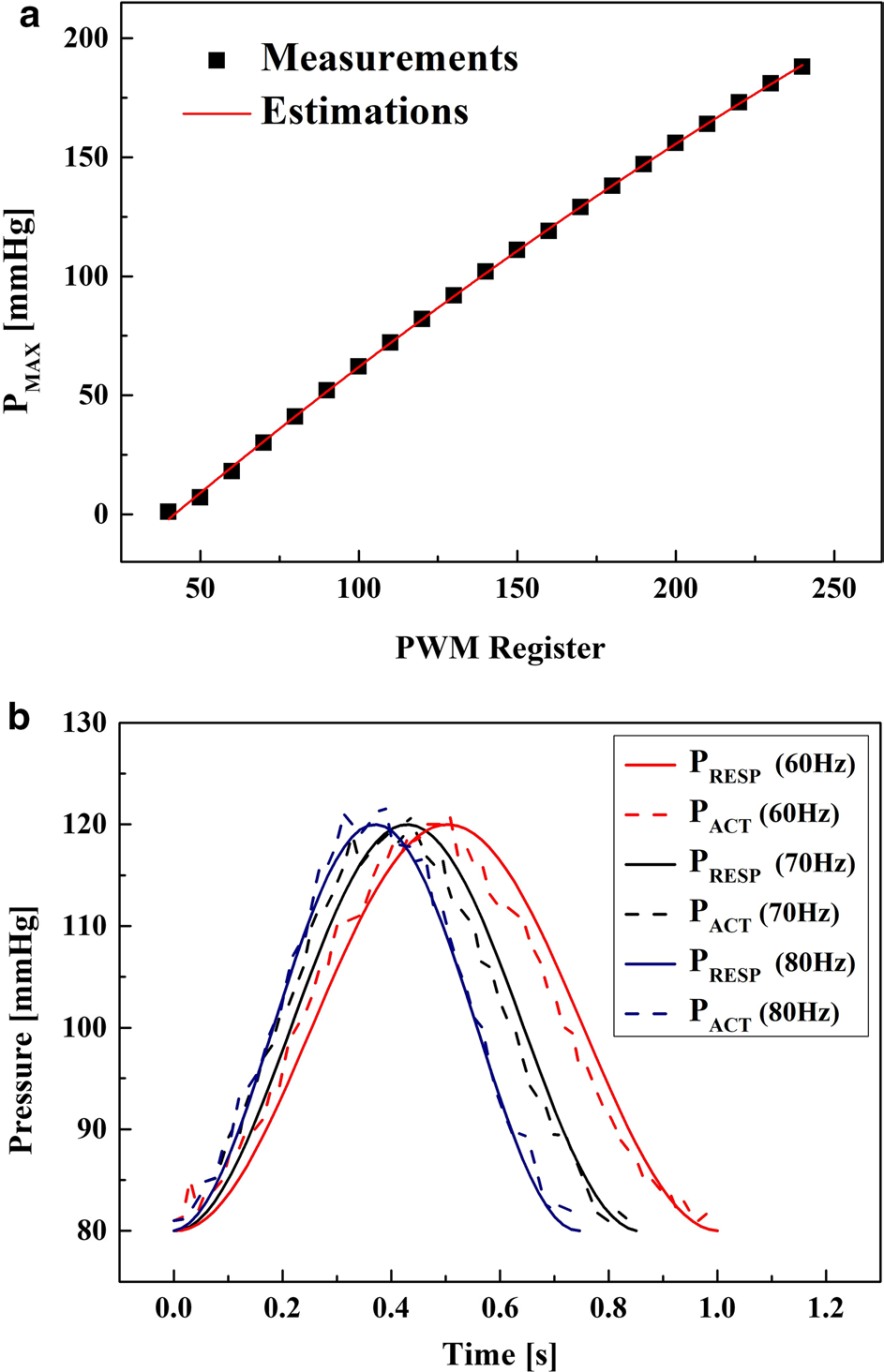
Results of the performance evaluation tests. **a** Comparison between the measurements and estimations of *P*_MAX_ when the PWM register was adjusted from 0 to 240. **b** Comparison between the calculations of *P*_RESP_ (solid lines) and the measurements of pressure sensor in the implemented model (dashed lines) at 60, 70, and 80 BPM while the reference systolic BP was fixed to 120 mmHg. *PWM* pulse width modulation, *P*_*ACT*_ measurements of pressure sensor

Figure 1a demonstrates the measurements and estimations of *P*_MAX_ when the value of PWM control register was adjusted from 0 to 240 with a step of 10 (*R*^2^ = 0.999 in Pearson correlation). Figure 1b demonstrates the reference *P*_RESP_ waveforms calculated by Eq. (2) (solid lines) and the measurements of the pressure sensor in the implemented model (dashed lines) when the PR_REF_ was adjusted to 60, 70 and 80 BPM while the SBP_REF_ was fixed to 120 mmHg. In the Pearson correlation analysis, the values of the Pearson coefficient were 0.983, 0.983, and 0.997 for the curves of 60, 70 and 80 BPM, respectively (*p* < 0.05 for all curves). Maximal errors between two curves were 4.9 BPM for 60 BPM, 4.7 BPM for 70 BPM, and 3.2 BPM for 80 BPM, respectively, and minimal errors between two curves were 0.1 BPM for 60 BPM, 0.0 BPM for 70 BPM, and 0.0 BPM for 80 BPM, respectively. Average errors between two curves were 2.3 BPM for 60 BPM, 2.3 BPM for 70 BPM, and 1.2 BPM for 80 BPM, respectively.

Table 1 shows the results of C1, C2 and C3 tests. From the recorded pressure waveforms, the points around each local maximum whose value is the largest were determined as the systolic BP points, and the time intervals between two adjacent systolic BP points were used to calculate the actual real-time PR. In C1 test, average errors between the reference and the measured SBP/PR were 1.2/0.7, 1.8/0.2, 1.7/1.6, 1.0/1.1, and 0.7/1.9 mmHg/BPM when the PR_REF_ was 60, 70, 80, 90 and 100 BPM, respectively. In C2 test, average errors between the reference and the measured SBP/PR were 0.4/1.6, 0.1/1.6, 0.2/1.6, 0.9/1.6, and 1.9/1.6 mmHg/BPM when the SBP_REF_ was 90, 100, 110, 120 and 130 mmHg, respectively. In C3 test, average errors between the reference and the measured SBP/PR were 2.4/0.7, 1.1/0.2, 1.2/1.6, 0.7/1.1, and 0.8/1.9 mmHg/BPM when the reference values of {SBP, PR} varied as {90, 60}, {100, 70}, {110, 80}, {120, 90}, and {130, 100}, respectively.

**Table 1:** Results of C1, C2 and C3 tests aimed to evaluate the performance of the implemented *P*_RESP_ control algorithm (mean ± standard deviation format)

C1 test			C2 test			C3 test		
Reference SBP/PR	Measured SBP/PR	Errors in SBP/PR	Reference SBP/PR	Measured SBP/PR	Errors in SBP/PR	Reference SBP/PR	Measured SBP/PR	Errors in SBP/PR
120/60	118.8 ± 0.6/59.3 ± 1.04	1.2 ± 0.6/0.7 ± 1.0	90/80	89.6 ± 0.49/78.4 ± 2.17	0.4 ± 0.5/1.6 ± 2.2	90/60	87.6 ± 0.49/59.3 ± 1.04	2.4 ± 0.5/0.7 ± 1.0
120/70	118.2 ± 0.4/69.8 ± 1.24	1.8 ± 0.4/0.2 ± 1.2	100/80	99.9 ± 0.54/78.4 ± 2.17	0.1 ± 0.5/1.6 ± 2.2	100/70	98.9 ± 0.83/69.8 ± 1.24	1.1 ± 0.8/0.2 ± 1.2
120/80	118.3 ± 0.45/78.4 ± 2.17	1.7 ± 0.5/1.6 ± 2.2	110/80	109.8 ± 0.87/78.4 ± 2.17	0.2 ± 0.9/1.6 ± 2.2	110/80	111.2 ± 0.4/78.4 ± 2.17	1.2 ± 0.4/1.6 ± 2.2
120/90	119 ± 0.77/88.9 ± 3.68	1.0 ± 0.8/1.1 ± 3.7	120/80	119.1 ± 0.54/78.4 ± 2.17	0.9 ± 0.5/1.6 ± 2.2	120/90	120.7 ± 0.6/88.9 ± 3.68	0.7 ± 0.8/1.1 ± 3.7
120/100	119.3 ± 0.46/98.1 ± 2.1	0.7 ± 0.5/1.9 ± 2.1	130/80	128.1 ± 0.54/78.4 ± 2.17	1.9 ± 0.5/1.6 ± 2.2	130/100	129.2 ± 0.6/98.1 ± 2.1	0.8 ± 0.8/1.9 ± 2.1

*SBP* systolic blood pressure (in mmHg), *PR* pumping rate (in BPM)

Table 2 shows the results of the usability test using ten BP monitors. Maximal average errors between the reference and the measured systolic BPs in three test conditions (in absolute value) were 1.8, 0.9, 1.5, 1.3, 2.8, 2.5, 0.8, 1.0, 2.9, and 0.9 mmHg at BPM-1, BPM-2, …, and BPM-10, respectively. Maximal average errors between the reference and the measured PRs in three test conditions (in absolute value) were 0.0, 1.0, 1.9, 0.8, 0.5, 1.3, 1.0, 1.7, 1.0, and 0.0 mmHg at BPM-1, BPM-2, …, and BPM-10, respectively. Maximal percentage errors between the reference and the measured PRs in three test conditions were 0.0, 0.7, 3.2, 1.3, 0.6, 2.2, 1.4, 2.8, 1.7 and 0.0% at BPM-1, BPM-2, …, and BPM-10, respectively.

**Table 2:** Results of the usability test of the implemented model using ten BP monitors (mean ± standard deviation format)

	Company/model	Type/mechanism	BP condition	Measurements/errors			
				SBP	Errors	PR	Errors
BPM-1	Inbody/BPBIO320	Stationary/inflationary oscillometry	HYPER	151.8 ± 2.6	1.8 ± 2.6	70	0
			NORMAL	121.4 ± 1.2	1.4 ± 1.2	60	0
			HYPO	79.8 ± 2.2	0.2 ± 2.2	80	0
BPM-2	AND/TM2655P	Stationary/deflationary oscillometry	HYPER	150.2 ± 2.0	0.2 ± 2.0	69.6 ± 0.6	0.4 ± 0.6
			NORMAL	120.9 ± 2.3	0.9 ± 2.3	59.7 ± 0.5	0.3 ± 0.5
			HYPO	79.5 ± 3.0	0.5 ± 3.0	79	1.0
BPM-3	PHILIPS/IntelliVue X2	Portable/deflationary oscillometry	HYPER	149.1 ± 2.5	0.9 ± 2.5	70.2 ± 1.1	0.2 ± 1.1
			NORMAL	119.4 ± 2.4	0.6 ± 2.4	61.9 ± 1.5	1.9 ± 1.5
			HYPO	81.6 ± 3.3	1.6 ± 3.3	79.1 ± 1.4	0.9 ± 1.4
BPM-4	PHILIPS/IntelliVue MP2	Portable/deflationary oscillometry	HYPER	150.4 ± 2.7	0.4 ± 2.7	69.8 ± 0.6	0.2 ± 2.3
			NORMAL	120.4 ± 2.3	0.4 ± 2.3	60.8 ± 1.3	0.8 ± 1.3
			HYPO	81.3 ± 1.8	1.3 ± 1.8	79.3 ± 1.3	0.7 ± 1.3
BPM-5	PHILIPS/IntelliVue M3001A	Portable/deflationary oscillometry	HYPER	152.7 ± 2.0	2.7 ± 2.0	69.9 ± 0.9	0.1 ± 0.9
			NORMAL	121.4 ± 2.2	1.4 ± 2.2	60.3 ± 0.9	0.3 ± 0.9
			HYPO	82.8 ± 1.6	2.8 ± 1.6	79.5 ± 1.1	0.5 ± 1.1
BPM-6	PHILIPS/IntelliVue MP5	Portable/deflationary oscillometry	HYPER	150.7 ± 2.7	0.7 ± 2.7	69.9 ± 0.8	0.1 ± 0.8
			NORMAL	122.5 ± 1.9	2.5 ± 1.9	61.3 ± 1.7	1.3 ± 1.7
			HYPO	81.6 ± 3.5	1.6 ± 3.5	79.7 ± 1.6	0.3 ± 1.6
BPM-7	PHILIPS/IntelliVue MP2	Portable/deflationary oscillometry	HYPER	150.8 ± 1.5	0.8 ± 1.5	69.0 ± 0.2	1.0 ± 0.2
			NORMAL	120.0 ± 0.9	0.0 ± 0.9	59.5 ± 0.5	0.5 ± 0.5
			HYPO	80.7 ± 0.9	0.7 ± 0.9	80	0
BPM-8	PHILIPS/IntelliVue X2	Portable/deflationary oscillometry	HYPER	150.6 ± 3.0	0.6 ± 3.0	71.4 ± 1.3	1.4 ± 1.3
			NORMAL	119.4 ± 2.5	0.6 ± 2.5	61.7 ± 1.0	1.7 ± 1.0
			HYPO	81.0 ± 3.4	1.0 ± 3.4	79.6 ± 1.1	0.4 ± 1.1
BPM-9	MEDIANA/M20	Portable/deflationary oscillometry	HYPER	151.8 ± 2.6	1.8 ± 2.6	69	1
			NORMAL	117.3 ± 2.5	2.7 ± 2.5	59	1
			HYPO	77.2 ± 2.1	2.8 ± 2.1	79	1
BPM-10	MEDIANA/M30	Portable/deflationary oscillometry	HYPER	149.1 ± 3.2	0.9 ± 3.2	70	0
			NORMAL	119.3 ± 1.4	0.7 ± 1.4	60	0
			HYPO	77.5 ± 2.8	2.5 ± 2.8	80	0

*SBP* systolic blood pressure (in mmHg), *PR* pumping rate (in BPM), *HYPER* hypertension, *NORMAL* normal, *HYPO* hypotension

Table 3 shows the measurements before and after the intentional performance deterioration (abnormal elevation of the cuff pressure measurement) of the selected BP monitor (BPM-2) using the proposed model. Before deterioration, average errors between the reference and the measured SBP/PR were 0.2/0.5, 0.9/0.4, and 0.5/1.0 mmHg/BPM when the test condition was HYPER, NORMAL, and HYPO, respectively. On the contrary, after intentional performance deterioration, those were 14.9/0.9, 15.2/0.2, and 15.6/0.8 mmHg/BPM. In the independent t-test, there were statistically significant differences in the systolic BP between two groups in all test conditions (*p* < 0.05); in contrast, there was no significant difference in the PR between two groups in HYPER and NORMAL conditions (*p* > 0.05). In HYPO condition, there was a statistically significant difference in the PR between two groups because the value of standard deviation was always zero before performance deterioration (*p* < 0.05).

**Table 3:** The measurements and errors before and after the intentional performance deterioration of the selected BP monitor (BPM-2) using the implemented model (mean ± standard deviation format)

	Before		After	
	SBP/PR	Errors in SBP/PR	SBP/PR	Errors in SBP/PR
HYPER	150.2 ± 2.0/69.6 ± 0.6	0.2 ± 2.0/0.4 ± 0.6	164.9 ± 1.3/69.1 ± 0.2	14.9 ± 1.3/0.9 ± 0.2
NORMAL	120.9 ± 2.3/59.6 ± 0.5	0.9 ± 2.3/0.3 ± 0.5	135.2 ± 1.3/59.8 ± 0.4	15.2 ± 1.3/0.2 ± 0.4
HYPO	79.5 ± 3.0/79	0.5 ± 3.0/1.0	95.6 ± 1.9/79.2 ± 0.4	15.6 ± 1.9/0.8 ± 0.4

*SBP* systolic blood pressure (in mmHg), *PR* pumping rate (in BPM), *HYPER* hypertension, *NORMAL* normal, *HYPO* hypotension

## Discussion

The purpose of this study is not making a new inspection device that assesses the accuracy of BP monitor; e.g., BP PUMP2 (Fluke biomedical, Washington, USA), AccuPulse (Clinical Dynamics, Wallingford, USA), AccuSim-BP (Datrend Systems Inc., Richmond BC, Canada), SC-5 SimCube (Pronk Technologies, Inc., Sun Valley, USA), and MS200 (Contec Medical Systems Co., Ltd., Qinhuangdao, China) [4]. For this purpose, it is mandatory to use an inspection device that passed technical guidelines of regulatory communities and well-designed clinical verifications, which is far from our current technical model [5, 6]. Our target was just to implement a technical model that is suitable for quick and simple on-site pre-screening test to detect error-suspicious devices among plural in-hospital BP monitors before time-consuming official inspection in the engineering department. This kind of device may not so beneficial for consumer-selling vendors and small-scale hospitals; on the contrary, it can be useful for most large-scale hospitals that equip plural (tens or hundreds) in-hospital BP monitors at various sites but weekly or monthly inspection of each device is not possible. For these hospitals, for example, more improved management protocol can be possible using the proposed technical model as follows. First, the pseudo-BP generator is equipped in sites (e.g., wards, outpatient clinics, and emergency room) where the BP monitor is utilized. Second, nurses perform on-site pre-screening test by themselves using the equipped pseudo-BP generator weekly or monthly. Third, only when one or more BP monitors are suspicious during the on-site tests—e.g., the pseudo-BP generator is set to 100 mmHg SBP but the measurements of BP monitor are consistently over 110 mmHg or under 90 mmHg—, the nurse calls the engineering department to request further inspection.

Compared to conventional inspection devices, the proposed technical model has several advantages as follows. First, the evaluation protocol is similar to the normal BP measurement; so, nurses can easily understand how to use it. Second, it is suitable for frequent on-site inspections because it is small, light, quick, single-pieced, and non-hydraulic. Third, its manufacturing cost is relatively low; thus making it possible to equip at various sites simultaneously with less financial burden. Besides, as shown in Table 2, the proposed model demonstrated an almost even evaluation performance for BP monitors with different models and vendors. This versatility is especially important for large-scale hospitals that furnish plural BP monitors with various models and vendors.

There have been a few reports that proposed cuff-insertion type BP monitor inspection devices. For example, Yong and Geddes proposed a surrogate arm that requires inserting a plastic cylindrical chamber that contains air/water mixture into the cuff [7]. However, for this device to inspect the BP monitor, additional components, such as an external water-bath, a water-filled balloon, and an air source, should be attached to the cuff-inserted chamber, which makes the overall system large, heavy, and bulky. In addition, water supply and water discharge are necessary before and after the inspection; therefore, it is not suitable for quick and convenient on-site inspections. In addition, Kim et al. proposed an arm-type BP simulator that utilizes pneumatic pressure, not hydraulic pressure [8]. However, they used a bellows, servo disk motor, and screw piston for pneumatic pressure generation; therefore, it is difficult to manufacture a small and light single-pieced device, and as a result, it is not suitable for on-site inspections, too. Compared to these reports, our model does not require any large, heavy and bulky hydraulic components and can be manufactured as totally single-pieced, which improves examiner convenience.

Under the general regulatory guidelines for BP monitor, clinically permissible ranges of measurement error are (1) maximal average error in BP measurement ≤ 5 mmHg, (2) maximal standard deviation in BP measurement ≤ 8 mmHg, and (3) maximal average error in PR measurement ≤ 5%, respectively [9–11]. As shown in Table 2, the maximal error in average SBP measurement was 2.9 mmHg (HYPO in BPM-9), the maximal standard deviation in average SBP measurement was 3.5 mmHg (HYPO in BPM-6), and the maximal percentage error in average PR measurement was 3.2% (NORMAL in BPM-3); that is, all of the three parameters satisfied the requirements of guidelines. Although more detailed verification processes, such as in vitro test, animal experiments, and clinical trials, are required in future studies, the current experimental results might show the potential of the proposed model as a BP monitor inspection device.

The limitations of the current study are as follows. First, as described above, the main purpose of the study was to make a single-pieced, fully air-driven, cuff-inserted technical model that can be used only for easy and simple on-site pre-screening purpose to detect error-suspicious devices before time-consuming official inspection. Therefore, we used a simple sinusoidal wave-profile as a reference to simplify the implementation, and fixed the difference between the SBP_REF_ and DBP_REF_ as 40 mmHg based on the assumption that when the accuracy of an embedded pressure sensor in the BP monitor deteriorates, both of the SBP and DBP measurements will become inaccurate simultaneously. Second, the current model cannot be applied to assess the accuracy of BP monitors directly because the operating mechanism of the proposed model is far from a standard approach of the conventional BP monitor inspection devices, and as a result, it should be verified through more well-designed validation processes in future studies. Third, there have been two main approaches for benchtop assessment of BP monitors—i.e., limb simulators and waveform generators—and it is generally known that the latter is more successful than the former [12, 13]. Although the current model showed reasonable performance during the experiments, details of the current model need to be more optimized for reliable and reproducible measurements. For example, (1) physical properties of the air-tubes and elastic meshes should be more matched to those of actual brachial artery and skin, and (2) the pseudo-BP waveform of the model should be more matched to that of actual limb.

## Conclusions

In this study, we proposed a single-pieced, fully air-driven, cuff-inserted technical model for easy, simple, and quick on-site BP monitor pre-screening purpose before official inspection, and evaluated the performance and versatility of a proposed model using ten BP monitors. We expect that the proposed model can give a technical option to improve the quality of BP monitor management and the safety of patients with abnormal BP especially for large-scale hospitals.

## Methods

### Implementation of a single-pieced, fully air-driven pseudo-BP generator

**Fig. 2:**
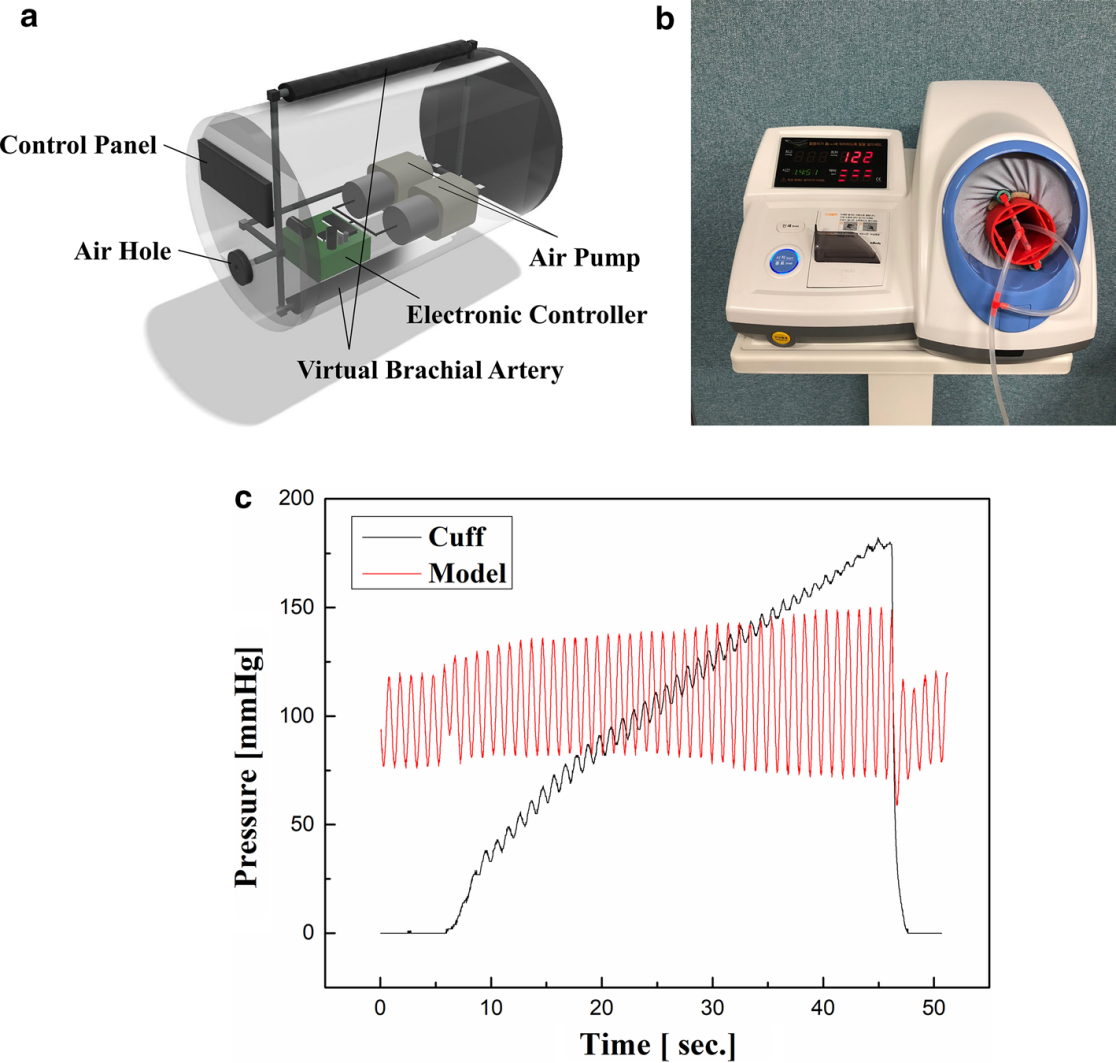
Schematics of the proposed model for pseudo-BP generation. **a** The internal structure of the model, **b** inserting the implemented model into the cuff of BP monitor, **c** the waveforms of model-generated pressure (red line) and cuff pressure (black line) during the measurement when the model was set to 120 mmHg systolic BP and 60 BPM PR

In standard oscillometric BP measurement, a BP monitor detects the vibration of cuff pressure due to the occlusion and re-opening of brachial artery in the cuff during the measurement, and then, calculates average, systolic and diastolic BPs and HR [14]. To simulate this circumstance, we designed a single-pieced, fully air-driven pseudo-BP generator as Fig. 2a. Two flexible air-tubes that simulate brachial artery (diameter = 2.5 cm, length = 19 cm) are arranged outside the rigid cylinder (diameter = 7 cm, length = 22 cm) with 180° spacing. To fix the position of these air-tubes during BP measurements, elastic meshes (CN03; Winner Industries Co., Ltd, Shenzhen, China) are inserted between each air-tube and the rigid cylinder, and the upper side of the air-tubes is covered by the same elastic mesh that is pulled with a constant force of about 8 N while winding. In the rigid cylinder, an electronic controller, two air-pumps (KPM32E; Koge Micro Tech Co., New Taipei City, Taiwan), and a pneumatic pressure sensor (MPX5100GP; NXP Semiconductors N.V. Inc., Eindhoven, Netherlands) are placed to adjust the values of upper peaks (denoting systolic BP) and frequency (denoting HR) of the pseudo-BP waveform. The generated pseudo-BP is then transferred to the cuff of the BP monitor through the air-tubes inserted into the cuff. Two air pumps, two air-tubes, a pneumatic pressure sensor, and an air-hole are connected together via polymer tubes and plastic connectors to construct a single closed air-loop. During the implementation, the cylinder and connectors were manufactured using a 3D printer (Zortrax M200; Zortrax Corp., Olsztyn, Poland). For example, to test a certain BP monitor with inflationary oscillometry mechanism, the implemented pseudo-BP generator is inserted into the cuff of the BP monitor and starts operation to generate a user-defined systolic BP and pumping rate (PR) conditions. Then, the BP monitor starts to compress the cuff-inserted pseudo-BP generator as normal BP measurement situations (Fig. 2b) until the cuff pressure reaches about 300 mmHg. Then, the BP monitor measures the vibrations of cuff pressure and calculates BP and HR conditions based on its own algorithm. Figure 2c shows the waveforms of model-generated pressure and cuff pressure during the measurement when the implemented pseudo-BP generator was set to generate a pressure waveform with 120 mmHg systolic BP and 60 beats/min (BPM) PR.

### Control algorithm to generate pseudo-BP waveforms through the air-tubes

**Fig. 3:**
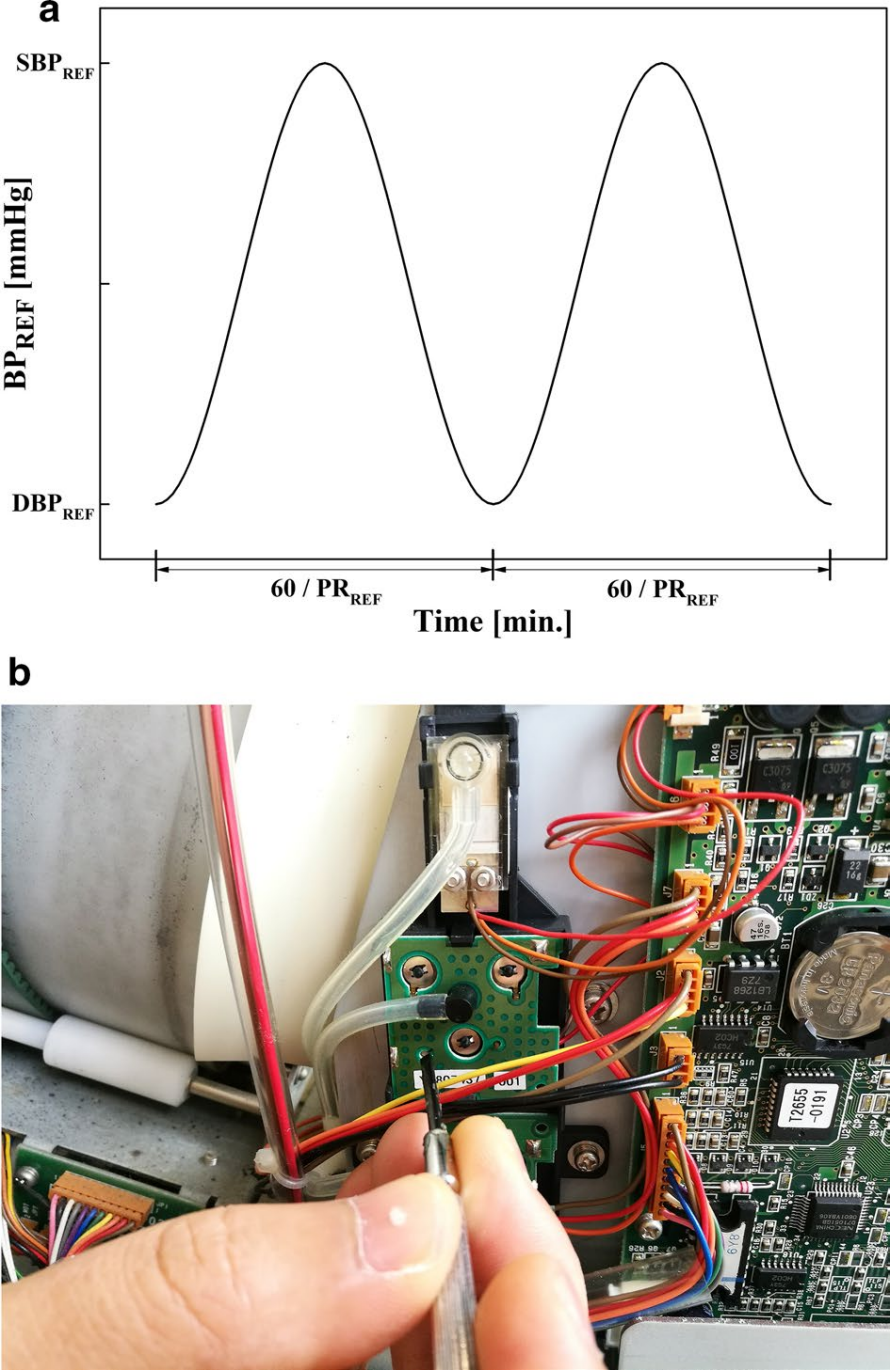
Implementation of the pseudo-BP generation algorithm. **a** Reference waveform of the pseudo-BP given the reference values of systolic BP, diastolic BP and PR; **b** adjusting the variable resistor in BPM-2 using a screwdriver to deteriorates the accuracy of BP measurement

First, we set the reference waveform of the generated pseudo-BP (BP_REF_) for given BP and PR conditions as Eq. (1) (Fig. 3a).

1 $${\text{BP}}_{\text{REF}} \left( {\text{mmHg}} \right) = \left( {\frac{{{\text{PR}}_{\text{REF}} }}{60}} \right)\left( {\frac{{{\text{SBP}}_{\text{REF}} - {\text{DBP}}_{\text{REF}} }}{2}} \right)\sin \left( {2\pi \left( {\frac{{{\text{PR}}_{\text{REF}} }}{60}} \right)t} \right) + \left( {\frac{{{\text{SBP}}_{\text{REF}} + {\text{DBP}}_{\text{REF}} }}{2}} \right),$$

where SBP_REF_, DBP_REF_ and PR_REF_, represent the reference values of systolic BP, diastolic BP and PR by the implemented model. In our experiments, the amplitude of the pneumatic pressure in the closed air-loop reduced to 1/*N* when the value of PR_REF_ was *N*-times increased due to the decrease of pressure restoration time when PR_REF_ increased; to compensate for this, {PR_REF_/60} was multiplied to the amplitude of the reference BP waveform in Eq. (1). Then, we further simplified Eq. (1) by assuming that the difference between the SBP_REF_ and the DBP_REF_ is always 40 mmHg. That is, when the examiner set the values of SBP_REF_ and PR_REF_, the amplitude of the BP_REF_ waveform was automatically set to 20 mmHg (i.e., half of the difference between the SBP_REF_ and the DBP_REF_). Then, the reference waveform moved upward to match the values of local minima with the {SBP_REF_—20 mmHg}, and the responsive pressure in the closed air-loop (*P*_RESP_) that corresponds to the given SBP_REF_ and PR_REF_ was calculated by Eq. (2).

2 $$P_{\text{RESP}} = \left( {\frac{{{\text{PR}}_{\text{REF}} }}{3}} \right) \times \sin \left( {2\pi \left( {\frac{{{\text{PR}}_{\text{REF}} }}{60}} \right)t} \right) + \left( {{\text{SBP}}_{\text{REF}} - 20} \right).$$

In the current study, we used an air-hole component whose hole-diameter is about 1 mm, and empirically determined the difference between the SBP_REF_ and the DBP_REF_ as 40 mmHg based on the actual *P*_RESP_ measurements from repetitive experiments. To generate the BP_REF_ based on Eq. (2), the electronic controller adjusts the peaks and rates of the pneumatic pressure waveform inside the closed air-loop by pulse width modulation (PWM) control. More specifically, we increased the value of 8-bit register that adjusts the duty ratio of PWM signal from 0 (duty ratio = 0.00%) to 240 (duty ratio = 93.75%) with a step of 10 (step = 3.91%). Then, the local maxima (*P*_MAX_) values of the generated pressure waveform at each duty ratio conditions were recorded using a pressure gauge (Heine Gamma G5; Heine Optotechnik GmbH & Co. KG, Herrsching, Deutschland) that is also connected to the closed air-loop. Then, the relationship between the duty ratio (in %) and the *P*_MAX_ (in mmHg) was extracted by applying the polynomial curve fitting, and finally, by substituting the *P*_RESP_ in Eq. (2) for the *P*_MAX_, the relationship between the duty ratio and the *P*_RESP_ was calculated as given by Eq. (3):

3 $${\text{Duty}}\;{\text{ratio }}\left( \% \right) \, = \, 0.000985 \times P_{\text{RESP}}^{2} + 0.861 \times P_{\text{RESP}} + \, 42.592.$$

### Evaluation of the performance of implemented model

In this implementation, the operating ranges of PR_REF_ and SBP_REF_ were restricted to 60–120 BPM and 80–150 mmHg, respectively. To evaluate the implemented *P*_RESP_ control algorithm, the values of *P*_RESP_ in Eq. (2) and measurements of the pressure sensor in the implemented model were compared each other in three test conditions. First, to verify the ability to adjust PR_REF_ while maintaining the constant SBP_REF_, (1) the value of SBP_REF_ was fixed to 120 mmHg, and (2) the value of PR_REF_ was adjusted from 60 to 100 BPM with 10 BPM step (denoted as C1 test). Second, to verify the ability to adjust SBP_REF_ while maintaining the constant PR_REF_, (1) the value of PR_REF_ was fixed to 80 BPM, and (2) the value of SBP_REF_ was adjusted from 90 to 130 mmHg with 10 mmHg step (denoted as C2 test). Third, to verify the ability to adjust both the SBP_REF_ and PR_REF_ simultaneously, (1) values of SBP_REF_ and PR_REF_ were initially set to 90 mmHg and 60 BPM, and (2) test condition {SBP_REF_ (mmHg), PR_REF_ (BPM)} was adjusted to {90, 60}, {100, 70}, {110, 80}, {120, 90}, and {130, 100} (denoted as C3 test). Each test condition was repeated ten times during the experiments.

Next, to evaluate the clinical usability of the proposed model, ten BP monitors that were used in our hospital were randomly selected as Table 2 (BPM-1 to BPM-10; two stationary and eight portable; one inflationary and nine deflationary). Then, the implemented model was applied to each of the selected BP monitors and was operated with three representative SBP/PR conditions [15]: {SBP_REF_, PR_REF_} = {150, 70} for hypertension (denoted as HYPER), {120, 60} for normal (denoted as NORMAL), and {80, 80} for hypotension (denoted as HYPO). All the tests were repeated 30 times during the experiments.

Next, to evaluate whether the proposed pseudo-BP generator can be used to discriminate the error-suspicious BP monitor whose measurement error is out of the permitted range, we opened the external case of an arbitrarily selected BP monitor (BPM-2) and adjusted the variable port in front of the embedded pressure sensor using a screwdriver to increase the velocity of air flow toward the sensor, which results in the elevation of the level of cuff pressure measurement (i.e., case of embedded sensor error; Fig. 3b). Then, the proposed pseudo-BP generator was applied to the modified BP monitor and the measurements at HYPER, NORMAL, and HYPO conditions were recorded 30 times each. Finally, the measurements before and after the intentional performance deterioration were compared with each other.

## Data Availability

The dataset supporting the conclusions of this article is included within the article.

## Abbreviations

BP: blood pressure
HR: heart rate
SBP: systolic blood pressure
DBP: diastolic blood pressure
PR: pumping rate
BPM: beats per minute
PWM: pulse width modulation
HYPER: hypertension
HYPO: hypotension
